# Acute Liver Injury, Rhabdomyolysis, and Acute Kidney Injury Following Mass Envenomation by Wasps in Malaysia

**DOI:** 10.7759/cureus.24369

**Published:** 2022-04-22

**Authors:** Neerusha Kaisbain, Mahaletchumi Rajappan, Wei Juan Lim, Chee Yik Chang

**Affiliations:** 1 Internal Medicine, Hospital Sultanah Aminah, Johor Bahru, MYS; 2 Cardiology, National Heart Institute (IJN), Kuala Lumpur, MYS; 3 Internal Medicine, Hospital Sungai Buloh, Selangor, MYS

**Keywords:** malaysia, wasps, : acute kidney injury, hepatocellular liver injury, mass envenomation, rhabdomyolysis

## Abstract

A wasp sting is not uncommon in rural areas, especially in developing countries. While severe allergic reactions to wasp sting are well known, many are unaware of its dangerous systemic toxic reactions. In addition, these systemic toxic reactions occur more gradually as compared to anaphylaxis reactions, which occur rapidly. These deadly systemic reactions can be deceiving, as the local reactions may seem benign and harmless. In an untrained eye with a low index of suspicion, these systemic toxic reactions may be missed without repeated laboratory evaluations and may prove fatal without the timely institution of supportive treatments.

## Introduction

Wasps, bees, and hornets are medically important insects of the order Hymenoptera [1-2]. Wasp stings are well known to cause allergic reactions, ranging from mild reactions to severe anaphylactic reactions [3]. It can occur after a single wasp sting, especially in a previously sensitized individual [3]. But rarely, it can cause systemic toxic reactions, which usually occur after mass envenomation by a swarm of wasps. The kidneys are one of the most commonly affected organs [1-3]. There are multiple case reports describing acute kidney injuries requiring renal replacement therapies as a result of mass envenomation of wasps [1-4]. We, on the other hand, would like to report a case of wasp sting that was complicated by severe rhabdomyolysis and transaminitis predominantly, as well as mild kidney injury.

## Case presentation

**Figure 1:**
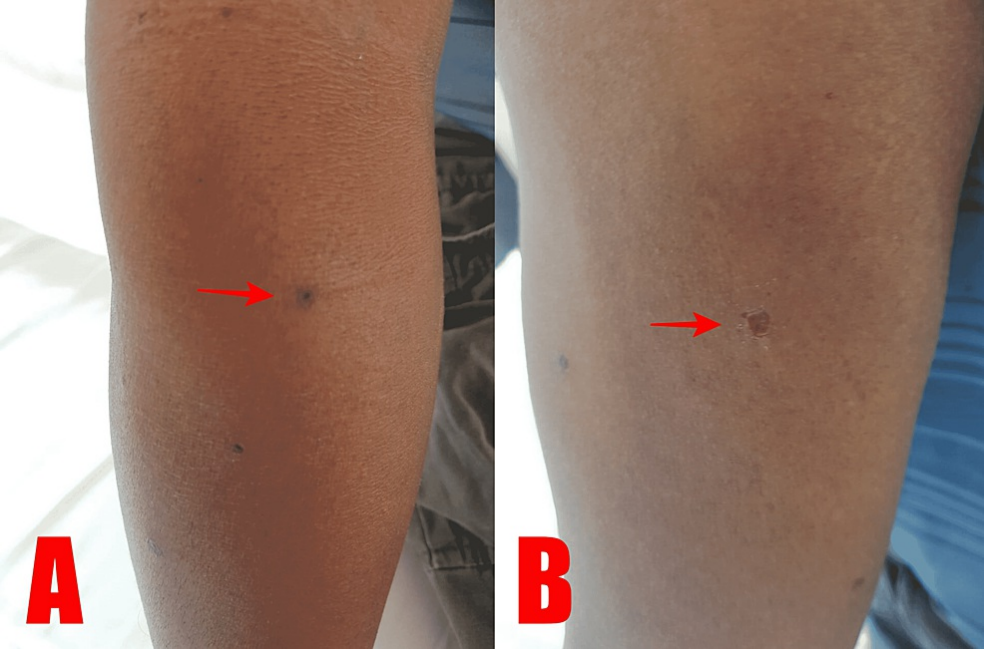
Showing wasp sting (red arrow) on the patient’s right arm (A) and forearm (B) taken on day three of the incident.

A 47-year-old gentleman with an unremarkable past medical history was stung by more than 10 wasps while he was plucking fruits, with the majority of the stings on his scalp and a few on his arms. He began to experience pain and swelling in his scalp. The patient experienced gastroenteritis-like symptoms on the first day of the incident, with more than 10 episodes of diarrhea and two to three episodes of vomiting. He denied any other symptoms such as chest pain, myalgia, or cutaneous manifestations of an allergic reaction. He did not report any previous history of a wasp sting. Clinically, he was calm and alert. There were a few sting marks over his arms (Figure 1) and the rest were embedded within his scalp. The local reactions were mild. The patient remained hemodynamically stable.

He presented to a district hospital and the blood investigations showed mild acute kidney injury and mild rhabdomyolysis as shown in Table 1. Unfortunately, the liver function test was not obtained on presentation as the liver injury was not suspected. He was admitted to the district hospital and was started on two liters of normal saline hydration per day. Blood investigations were repeated the following day, which showed a marked increment in the liver enzymes and creatinine kinase as shown in Table 1. He developed severe rhabdomyolysis and transaminitis with hyperbilirubinemia. Urine microscopic examination showed blood 3+, protein 3+, urobilinogen trace, and others were negative. However, he reported neither hematuria nor oliguria. Electrocardiogram revealed sinus rhythm with no ST-T changes. His hydration was increased to three liters per day and he was subsequently transferred to a tertiary hospital.

**Table 1:** Patient’s serial blood investigation results.

	DAY 1	DAY 2	DAY 3	DAY 4	DAY 5	DAY 14
Hemoglobin (g/L)	166		157		154	
White blood cell (x10^9/L)	16.6		16.1		9.1	
Absolute neutrophil count (x10^9/L)			12.2		5.0	
Absolute lymphocyte count (x10^9/L)			2.3		2.5	
Eosinophil count (x10^9/L)			0.8		0.9	
Platelet (x10^9/L)	309		223		230	
Sodium (mmol/L)	141	137	139	138	138	137
Potassium (mmol/L)	3.4	4.0	3.08	3.68	3.85	4.0
Urea (mmol/L)	7.1	7.8	3.6	3.5	3.9	4.5
Creatinine (umol/L)	112	110	81	86	81	100
Bilirubin (umol/L)		50	15.2	11.1	10.1	7
Alkaline phosphatase (U/L)		101	81	84	78	74
Alanine transaminase (U/L)		664	436	307	251	43
Aspartate transaminase (U/L)	380	2275	1132	526	259	35
Creatinine kinase (U/L)	412	40994	48717	18565	5875	93
Lactate dehydrogenase (U/L)	418	3139	2079	1234	819	215
Prothrombin time (s)			17.1	12.7		
Activated partial thromboplastin time (s)			55.2	36.5		
International normalized ratio			1.59	1.18		

On admission to the tertiary hospital, a coagulation profile was obtained which showed mild coagulopathy. Being in a country endemic to leptospirosis, he was tested for leptospirosis and his leptospira immunoglobulin M (IgM) was negative. Urine myoglobin sent two days after the presentation was negative. A highly sensitive Troponin T (hsTropT) taken on day three of the event was 9 ng/L, which was within normal range. His hepatitis B surface antigen and hepatitis C antibody were negative as well. Ultrasonography of his hepatobiliary system showed no focal liver lesions. He remained asymptomatic, despite a rising creatinine kinase trend.

He received IV fluid hydration with three liters of normal saline per day and maintained good urine output. By day three of the event, his renal function normalized and liver functions showed an improving trend. By day five, there were drastic improvements in his liver enzymes and creatinine kinase level with IV fluid hydration. He was discharged from the hospital on day five and was encouraged to take an adequate amount of fluids. Throughout his hospital stay, he remained hemodynamically stable. A follow-up review on day 14 showed normalization of the blood parameters as shown in Table 1.

## Discussion

Bees, wasps, and hornets, for example, have stings and it is actually an ovipositor (egg-laying organ) modified as a weapon to protect themselves and their colonies [2]. Males do not have an ovipositor. Thus, only females sting. Among these Hymenoptera, hornet stings are described to be three times more life-threatening than those of bees and wasps [3]. Wasps will sting if disturbed, and if they sensed a threat to their colony, hundreds of wasps may sting the offender, causing mass envenomation [4]. A wasp sting is not uncommon, especially in developing countries such as Bangladesh, India, Malaysia, and Indonesia.

When a person is stung by a Hymenoptera, their venom is introduced into the person’s skin [3]. Their venom is a concentrated mixture of various biogenic amines, such as kinins, phospholipases, hyaluronidase, histamines, acid phosphatases, etc., and they have direct and indirect hemolytic, neurotoxic, myotoxic, nephrotoxic, hepatotoxic, and vasoactive properties [3, 5-6]. The responses to Hymenoptera stings are classified as normal local reactions, large local reactions, systemic anaphylactic reactions, systemic toxic reactions, and unusual reactions [7-9]. Local reactions to wasp sting include pain and swelling [10]. Systemic allergic reactions can be mild, moderate (angioedema and bronchospasm), or severe (anaphylactic shock or laryngeal edema) [10]. The allergic manifestation of a wasp sting is well recognized by most, but there are numerous other systemic reactions that have been reported worldwide, namely, rhabdomyolysis, acute kidney injury, acute tubular necrosis, hemolysis, centrilobular necrosis of the liver, disseminated intravascular coagulation, subendocardial necrosis, intracerebral hemorrhage, etc. [5]. There is also a case report of posterior reversible encephalopathy syndrome occurring in Malaysia, acute pancreatitis occurring in Sri Lanka, and myocardial infarction post anaphylaxis to wasp sting ascribed to Kounis syndrome or allergic angina [11-13].

There are numerous case reports of wasp stings causing acute kidney injury and rhabdomyolysis. Rhabdomyolysis is likely due to the direct toxic effects of the venom on the muscles [14]. Its mechanism of kidney injury, on the other hand, is thought to be mainly due to acute tubular necrosis secondary to rhabdomyolysis (myoglobinuria) and intravascular hemolysis (hemoglobinuria) [4]. Other mechanisms include direct nephrotoxicity by the venom, hypotension caused by anaphylaxis reaction, etc. [4,15].

A single wasp sting can cause immunoglobulin E (IgE)-mediated anaphylaxis reaction, especially in previously sensitized persons, whereas, mass envenomation produced by multiple wasp stings can cause systemic reactions of toxin-mediated cellular damage [5-6]. Toxic effects of wasp venom and the release of inflammatory mediators such as interleukin-6 are believed to cause multiorgan dysfunction syndrome [14]. The severity of clinical manifestation is related to the number of stings [9]. In an analysis of 1091 cases of wasp stings in China by Ittyachen et al., they found that the laboratory values are found to be much more elevated in patients with ≥ 10 stings than those with < 10 stings [9]. The in-hospital mortality in the ≥ 10 stings groups was five times higher than that of the <10 stings group [9]. In a study by Vikrant and Parashar [16], 10-200 wasp stings can result in acute kidney injury or even death.

There is no antivenom available for wasp sting and treatment is mainly supportive [4]. Immediate management is pivotal, starting with treatment of anaphylaxis with adrenaline, steroids, and antihistamines, followed by recognition of toxin-related complications [3, 6]. The mainstay of treatment is adequate hydration [6]. This can prevent volume depletion, tubular obstruction, and aciduria, which may cause acute kidney injury [3, 6].

As mentioned, wasp stings can be life-threatening. Immediate death from wasp sting typically results from hypotension, laryngeal edema, or bronchial constriction within one hour [9]. Less commonly, immediate death occurs from toxic effects of mass envenomation of hundreds of wasp stings [9]. Again, in the analysis by Ittyachen et al., most died of multiorgan dysfunction syndrome after a period of hospitalization, non-anaphylactic shock, or other complications due to severe intoxication and only a small number of them died of anaphylactic shock [9].

Our patient achieved complete renal recovery without requiring renal replacement therapy, likely due to early hospitalization and aggressive hydration. His predominant systemic manifestations were severe rhabdomyolysis and transaminitis complicated with mild coagulopathy. Timely intervention with adequate hydration was instituted and he, fortunately, was able to make full recovery. What is intriguing is the seemingly benign mild local reactions. These were actually the entry points for the potentially fatal venoms of the wasp.

## Conclusions

In conclusion, patients with wasp stings need to be monitored closely. While anaphylaxis appears rapidly, making it easy to recognize and treat, other systemic toxic reactions such as rhabdomyolysis may be insidious in onset. The treating team needs to be cautious and order repeated laboratory evaluations. Late diagnosis and treatment can lead to increased morbidity and even death. 
